# A randomised controlled trial on the effects of a structural education module among women with polycystic ovarian syndrome on nutrition and physical activity changes

**DOI:** 10.1186/s12905-022-01861-4

**Published:** 2022-07-06

**Authors:** Sareh Dashti, Habibah Abdul Hamid, Suriani Mohamad Saini, Maiza Tusimin, Maimunah Ismail, Ali Jafarzadeh Esfehani, Siew Mooi Ching, Kai Wei Lee, Norzian Ismail, Jie Lin Wong, Latiffah Abdul Latiff

**Affiliations:** 1 Department of Community Health, Faculty of Medicine and Health Sciences, Universiti Putra Malaysia, 43400 Serdang, Selangor Malaysia; 2 Department of Obstetrics and Gynaecology, Faculty of Medicine and Health Sciences, Universiti Putra Malaysia, 43400 Serdang, Selangor Malaysia; 3 Department of Imaging, Faculty of Medicine and Health Sciences, Universiti Putra Malaysia, 43400 Serdang, Selangor Malaysia; 4 Department of Professional Development and Continuing Education, Faculty of Educational Studies, Universiti Putra Malaysia, 43400 Serdang, Selangor Malaysia; 5 Metabolic Research Center, Mashhad University of Medical Sciences, Mashhad, 9177948564 Iran; 6 Department of Family Medicine, Faculty of Medicine and Health Sciences, Universiti Putra Malaysia, 43400 Serdang, Selangor Malaysia; 7 Department of Medicine, Faculty of Medicine and Health Sciences, Universiti Putra Malaysia, 43400 Serdang, Selangor Malaysia; 8 Department of Pre-Clinical Sciences, Faculty of Medicine and Health Sciences, Universiti Tunku Abdul Rahman, 43000 Kajang, Selangor Malaysia

**Keywords:** PCOS, Woman, Education module, Intervention, Physical activity, Polycystic ovarian syndrome, Nutrition, Knowledge

## Abstract

**Background:**

Polycystic ovarian syndrome (PCOS) is a complex metabolic, endocrine and reproductive disorder that has a huge impact on the life of women. To ascertain the effectiveness of health education module among women with PCOS.

**Methods:**

This single-centre, randomised controlled trial was conducted on female staff of the University Putra Malaysia who were diagnosed with PCOS. Subjects were randomly assigned into intervention (n = 34) and control group (n = 35). In the intervention group, they need to take part in 8 education sessions in total over 6 months, and feedback was collected at the end of the session.

**Results:**

Primary outcome was changes in knowledge, attitude and practise of nutrition. Secondary outcomes were eating attitude and behaviour as well as knowledge, attitude and practise towards physical activity. After 6-months of intervention, there was a significant difference observed in nutrition knowledge 1 score (*p* < 0.001) and nutrition knowledge 2 score (*p* = 0.01) between intervention and control groups. Similarly, there was a significant difference observed in international physical activity questionnaire score (*p* = 0.02) between intervention and control groups. However there was no significant changes for attitude and practice of nutrition, eating attitude as well as knowledge, attitude and practise of physical activity.

**Conclusions:**

Our study showed that 6-months of education intervention can improve nutrition and physical activity knowledge. Based on this study, the education module may be considered an effective intervention for women with PCOS.

*Trial registration*: Name of the registry: Australian New Zealand Clinical Trials Registry (ANZCTR). Trial registration number: ACTRN12617000135314. Date of registration: 24/01/2017. URL of trial registry record: https://www.anzctr.org.au/Trial/Registration/TrialReview.aspx?id=372037

## Key messages

- A structured intervention based on information motivation and behaviour change model was successful in improving knowledge about nutrition and physical activity in PCOS women.
- Physical activity behaviour increased due to the performance sessions but leisure time physical activity remained unchanged in PCOS women in the intervention group.
- All methods were carried out in accordance with relevant guidelines and regulations in ethical approval and consent to participate section.

## Background

Polycystic ovarian syndrome (PCOS) is a combination of metabolic, endocrine and reproductive disorders [1–3]. Approximately 5–12% of the female population worldwide have been diagnosed with PCOS [1–3]. Irving Stein and Michael Leventhal described PCOS in 1935 as enlarged polycystic ovaries that were often accompanied by amenorrhea and hirsutism [4]. The spectrum of PCOS symptoms were identified in the past two decades. Classic presentations of PCOS include clinical or biochemical signs of increased serum androgen levels; menstrual abnormality in the form of amenorrhea, oligomenorrhea, or anovulation, infertility, hirsutism, as well as overweight or obesity. Women with PCOS are prone to cardiovascular disease, obstructive sleep apnea, hyper insulinemia and impaired glucose metabolism; and metabolic syndrome. PCOS has a dramatic impacts on women, especially due to infertility, cardiometabolic disease, and their psychological complications [5–8].

A structured education module will help patients to have a better understanding of their condition and proceed with lifestyle modification [8, 9]. The most appropriate means of generic and specific interventions to support attitude and behaviour change at population and community levels [10].

As little as 5% to 14% weight loss is linked to improvements in CVD factors, reducing abdominal fat, blood glucose, blood lipids, IR, serum androgens and increasing menstrual cyclicity, ovulation, and fertility [11]. PCOS in pregnancy can increase the risk of maternal complications, including pregnancy-induced hypertension (PIH), preeclampsia (PE), gestational diabetes mellitus (GDM), preterm birth (PTB), and cesarean section, as well as fetal complications, including neonatal morbidity, prematurity, fetal growth restriction (FGR), and neonatal complications, including large for gestational age (LGA) and small for gestational age (SGA), Neonatal Intensive Care Unit (NICU) admission [12]. Therefore, aside from its cost-effectiveness, lifestyle management is recommended to be implemented as the first line in management of metabolic syndrome and that the intervention should consist of physician and non-physician health professionals, which may include dietician, a professional in health education or behavioural psychology [13, 14]. Our study aimed to evaluate the changes in knowledge, attitude and practise of nutrition and physical activity as well as the eating attitude among women with PCOS when given intervention in form of an educational module.

## Methods

We conducted a single-centre, randomized single blinded controlled trial that compared the effects of an educational module on nutrition and physical activity. This trial was approved by the Ethical Committee of University Putra Malaysia.

### Participants

Participants were recruited among female staff aged 18 to 49 years old.

Inclusion criteria were female university staff at childbearing age (18–49 years), being diagnosed with PCOS and willingness to participate in the study. Exclusion criteria were consumption of oral contraceptives, hormonal treatment or insulin-sensitizing agents for more than 4 weeks, abnormal thyroid findings, non-classical adrenal hyperplasia, having underlying conditions, including hyperprolactinemia, hypogonadotropic hypogonadism, premature ovarian failure, ovarian cysts or tumors, congenital adrenal hyperplasia, androgensecreting tumor, Cushing’s syndrome, uterine disorders, and chromosomal anomalies, pregnancy, Menopause, having physical disability.

A list of employees was obtained from the Human Resources Department. They were selected randomly by using random number generator software, OpenEpi (version 3.02). University departments were approached by the researchers and a written consent was obtained from the head of each department. Subjects were approached by the researchers. A written informed consent was obtained from each subject and a date was set for the measurements in case the subject was willing to participate in the study. A subject was randomly selected from the list of employees in the same department in case any of the selected employees refused to participate in the study. Researchers then approached subjects. Subjects were for eligibility by filling questionnaires and assessing anthropometric measurements. In the first stage, a total of 675 subjects were randomly chosen from the human resource department, 329 were suspicious to have features of PCOS and were visited by a gynaecologist specialist for further confirmation.

### Diagnosis of PCOS

The diagnosis of PCOS was made according to the revised 2003 Rotterdam ESHRE/ASRM consensus criteria and the recommendations from the international evidence-based guideline for the assessment and management of polycystic ovary syndrome along with the exclusion of related disorders [15, 16]. Identification of potential subjects was made using two methods. Subjects who had a positive history for PCOS or ovarian cysts were considered suspicious and were called for further clinical and laboratory assessments. In subjects without the history of PCOS or ovarian cysts, objective criteria for hyperandrogenism in PCOS (hirsutism, acne and menstrual irregularity) were obtained using questionnaires. Hyperandrogenism was identified as either oligomenorrhea, defined as eight or less menses per year [15], hirsutism, defined as a Ferriman–Gallwey score ≥ 8 [17], and presence of moderate to severe acne, defined as Global Acne Grading System (GAGS) score > 18 [18]. These criteria were used to identify the subjects that were suspected to have PCOS. These subjects were called for further costly diagnostic evaluations.

In the next stage, suspicious subjects were clinically re-examined by an experienced gynaecologist with 15 years of experience in clinical serves. Ovarian ultrasound scan was performed for suspicious subjects by a radiologist. Next, serum total and free testosterone levels were tested to assess hyperandrogenism. Sonographic diagnosis of PCO was confirmed upon the condition of having 12 or more follicular cysts, 2–9 mm in diameter and/or increased ovarian volume (> 10 ml) [15]. Hyperandrogenaemia was defined as serum androgen level above normal values [a total testosterone ≥ 2.94 nmol/L (88 ng/dL) [14]. In the end, 675 subjects were screened for PCOS and 86 were confirmed to have PCOS. Free and total testosterone levels were measured for all subjects to evaluate hyperandrogenism status. The cut-offs for hyperndrogenism based on free and total testosterone were 3 nmol/l and 32 pmol/l, respectively [19]. Thyroid function tests, including serum thyroid stimulating hormone (TSH), T3 and T4, as well as serum prolactin were evaluated in all subjects before recruitment in order to roll out common differential diagnoses.

### Sample size calculation

Sample size was calculated based on the difference in the mean response of matched pairs of 7.8 based on the study done by Anto Suji et al. [20]. Therefore, the sample size was calculated as 25 pairs of subjects considering type I error of 0.05 and type II error of 0.01. After considering non response rate of 40%, the final number needed in each group was 42.

### Study process

Sampling was made based on randomized block sampling design. First, university was divided into two regions based on the distance to the intervention location. Then blocks were created based on faculty location. A random allocation list consisting of blocks of two, based on faculty location, was used. The allocation ratio was 1:1. The random allocation sequence was generated by a person who did not participate in study intervention and sampling. The first subject in each block was assigned to intervention group and the following subjects were allocated to control and intervention groups based on their department location. Due to the nature of the study, the participants and researchers could not be blinded about allocation but the analysis was performed by a statistician who was blinded regarding the allocation.

The intervention group was then invited to attend 8 sessions of structured education. The study team did not provide any form of the medical treatment for PCOS to both intervention and control group. The control group received usual care, including education pamphlet regarding lifestyle modification guidelines and information sheet about PCOS and its complications.

### Intervention

The Health Education Module was prepared based on the information-motivation-behavioural Skill (IMB) model. Based on the IMB model information regarding PCOS, Metabolic syndrome (MS) and healthy lifestyle, including healthy eating and having an active lifestyle, were provided to the subjects to improve their knowledge about PCOS, MS, nutrition and physical activity. Furthermore, information regarding the skills to maintain a healthy lifestyle was provided to the subjects through the module. Study intervention included an introduction session with the aim of teaching participants evaluate and identify their strengths and weaknesses.

The following 8 sessions (each session lasted 30 min and was conducted in lecture format) provided information about types of weight management diets and appropriate food choices and mastering each diet.

Exercise intervention included 32 sessions (each session lasted 45 min in workshop format with practical presentations) providing information about identifying the benefits of exercise in PCOS, mastering different weight management exercises and familiarizing with the function of each exercise.

Motivation sessions consisted of two sessions on empowering participants to increase their motivation towards healthy eating and staying active in the form of lectures and activities (role playing).

One stress management session was conducted to educate participants how to identify ways to deal with stress, handling with stress and practicing ways to deal with stress in the form of lecture, discussion and role-playing activity.

Two sessions of self-discipline were conducted to provide participants with the tips on how to discipline themselves in the form of discussion.

Two sleep and rest information sessions were conducted to provide knowledge on the proper rest time, identifying the benefits of adapting adequate rest and practicing proper sleep time in the form of discussion.

Furthermore general nutritional tips and information on eating and food choices, healthy recipes, portion control were provided weekly to participants.

### Quality control of health education module

The education module was pre-tested for clarity of meaning, language and the flow of contents on five women. The education module was reviewed by an expert panel (two gynecologists, two physicians, and one post-doctoral researcher in the field of health education).

### Measurements

Demographic data including age, number of children, education and marital status, as well as lifestyle factors, including smoking habits, alcohol consumption or sporting activities, were collected using a self-administered questionnaire.

### Questionnaires

#### Nutrition knowledge, attitude and practice

Two nutrition knowledge questionnaires (25 items and 10 items) were used to measure nutrition knowledge on food groups, general nutrition knowledge, principle of energy balance and healthy diet. A 10-item questionnaire was designed to assess the attitude towards consumption of healthy diet, proper food choices and weight reduction. A 10-item practice questionnaire was also designed to assess the level of practice of subjects regarding nutrition pyramid, healthy choices and healthy eating. These questionnaires were mainly developed based on the module content and were used to assess the efficacy of module based on the IMB model.

#### Eating attitude test 26 (EAT-26)

Eating attitude of subjects was assessed using a 26-item eating attitude test (EAT- 26), which is an accepted standardized self-reports symptom eating disorder [21]. EAT-26 items are rated using a six-point scale based on how often the individual engages in specific behaviours. The questions may be answered: Always, Usually, Often, Sometimes, Rarely, and Never. The EAT-26 scores equal or above 20 indicate high concern regarding body weight or eating disorders while scores below 20 indicate low concern about body weight and lower probability of eating disorders [22]. This questionnaire has been validated and used in Malaysia using factor analysis and the internal consistency was reported to be 0.86 [23].

#### Three-factor eating questionnaire 18-item (TFEQ-R18)

The 21-item Three-Factor Eating Questionnaire (TFEQ-R21) [24] is a scale that measures three domains of eating behaviour: cognitive restraint (CR), uncontrolled eating (UE) and emotional eating (EE). A shortened 18-item version (TFEQ-R18) with a revised three-factor structure on the basis of CR (six items; the conscious restriction of food intake to control body weight or to promote weight loss), UE (nine items; the tendency to eat more than usual because of a loss of control over intake) and EE (three items; overeating during dysphoric mood states) was developed using data from severely obese participants. This questionnaire has been validated on 313 Malaysian adult workers using exploratory factor analysis and the internal consistency ranged between 0.66 and 0.87 using the Cronbach’s alpha [25].

#### International physical activity questionnaire (IPAQ)

The International Physical Activity Questionnaire (IPAQ) is a valid and specific tool to measure PA level [26]. IPAQ assesses the physical activity of subjects in the past 7 days. IPAQ generates qualitative and assessment of physical activity including low, moderate and highly active as well as providing the metabolic equivalents (MET) minutes per week (MET min/w) as a quantitative measurement of the amount of activity for subjects. The S-IPAQ has been previously used in studies on Malaysia population [27, 28]. The English and Malay version of S-IPAQ were used in this study.

#### Physical activity knowledge, attitude and practice

A validated questionnaire was utilized to assess physical activity knowledge, attitude and practise. A 10-item physical activity knowledge questionnaire was designed to measure the knowledge of the study subjects on the benefits and the required amount of physical activity for women. A 10-item questionnaire was designed to assess the attitude towards performing physical activity and active living. A 10-item practice questionnaire was also designed to assess the level of practice of subjects regarding physical activity requirements. These questionnaires were prepared based on the IMB model and the information provided in the module to assess the efficacy of the module.

### Systolic and diastolic blood pressure

The systolic and diastolic blood pressure of the subjects was evaluated using a sphygmomanometer after 20 min of rest and in sitting position.

### Data analysis

The per protocol approach was used to analyse the data. Subjects with participation rate of less than 70% were excluded from the analysis. Comparison of normally disturbed and non-normally distributed variables between cases and controls was performed using independent t-test and Mann–Whitney test, respectively. The chi-square test was used to compare the distribution pattern of categorical variables between case and control groups. Two-way repeated measure ANOVA was performed to evaluate the group, time and group * time effects. The main outcome was the time*group effect. The level of statistical significance was *p* < 0.05. Data analysis was performed using the Statistical Package for Social Sciences (SPSS) software version 16.

## Results

**Fig. 1:**
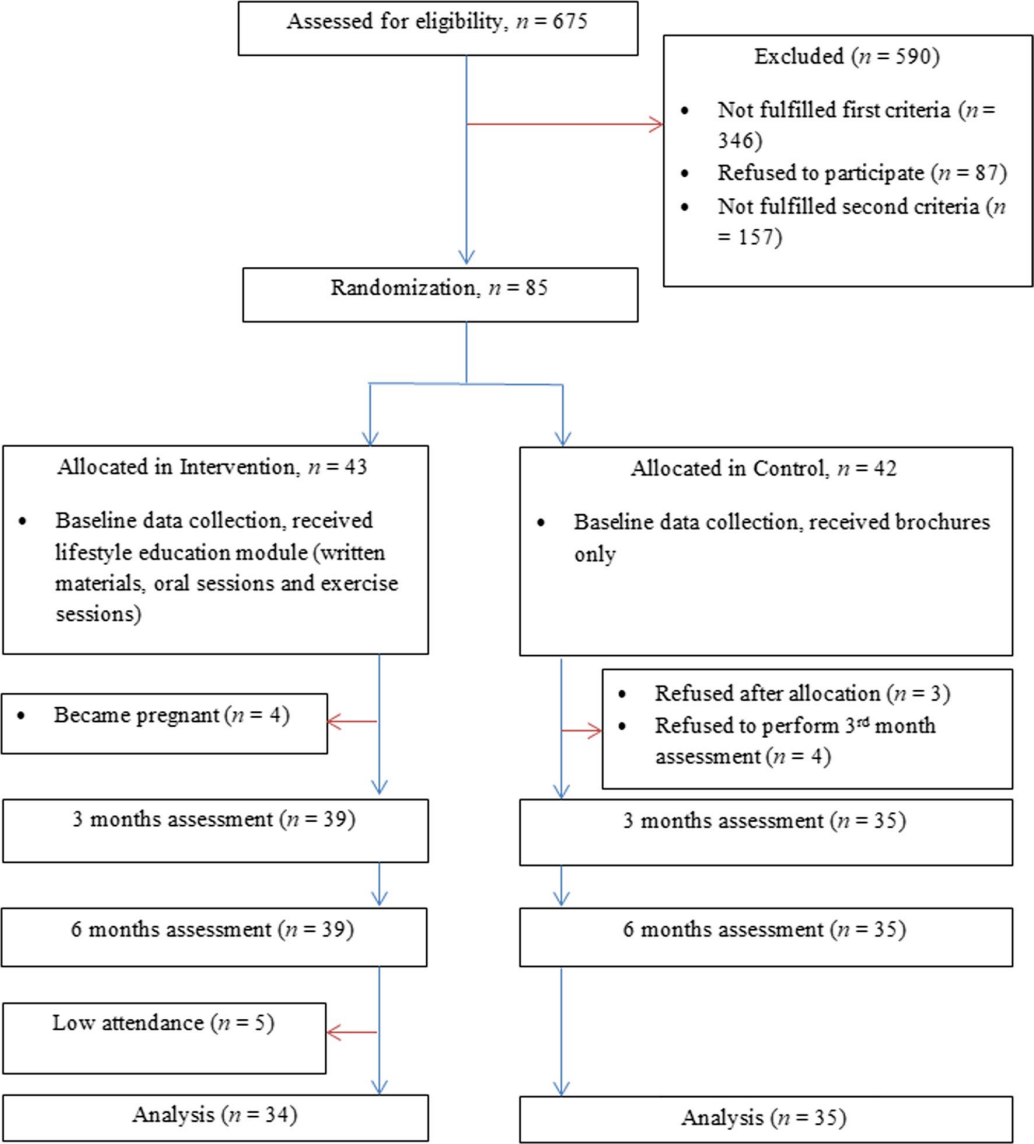
Consort diagram of participation in intervention

Consort diagram of participation in intervention is indicated in Fig. 1. Baseline characteristics of study subjects are shown in Table 1. A total of 329 (48.8%) out of 675 women, were found suspicious for PCOS based on the screening, while 37 (5.3%) were either previously diagnosed with PCOS (1, 0.1%) or had a past history of ovarian cysts without diagnosis of PCOS (36, 8.3%), which sum up to 366 (54.2%) suspicious subjects. Of the 245 subjects who were evaluated by radiologist, 85 were confirmed to have PCOS based on ultrasound scan. The 85 individuals were randomly assigned to intervention (43 subjects) and control (42 subjects) groups. Out of 42 subjects in the control group, 3 subjects refused to participate for baseline assessment (1 subject due to initiation of infertility medications which could influence weight and 2 subjects due to being randomized to control group and the need to wait for 6 months to receive the intervention module) and 4 subjects drop-out at 3rd month. The reason for drop-out in the intervention group was pregnancy in 4 (4.9%) of subjects. The reasons for drop-out among the control group included refusal to participate in the study after baseline assessment (3.7%) and loss to follow up in the 3^rd^ month assessment (4.9%). Pregnancy was defined as positive blood test for human chorionic gonadotropin obtained by a gynaecologist in suspicious cases.

**Table 1:** Description of study variables at baseline as per intervention groups and their comparison between groups

	Total (n = 82) Mean ± SD	Intervention group (n = 43) Mean ± SD	Control group (n = 39) Mean ± SD	t	*p*
Age (years)	32.05 ± 6.09	32.24 ± 6.51	31.86 ± 5.72	0.71	0.48
Weight (kg)	73.43 ± 17.94	74.09 ± 16.69	72.75 ± 19.36	071	0.48
Height (cm)	155.71 ± 5.04	155.45 ± 5.42	155.98 ± 4.68	− 0.14	0.89
BMI (kg/m^2^)	30.17 ± 6.73	30.46 ± 5.81	29.87 ± 7.62	0.71	0.48
Waist circumference (cm)	90.73 ± 13.64	90.65 ± 13.35	90.81 ± 14.11	0.15	0.88
Hip circumference (cm)	108.84 ± 13.46	108.24 ± 13.77	109.46 ± 13.30	− 0.26	0.75
Arm circumference (cm)	31.36 ± 5.00	31.74 ± 4.43	30.97 ± 5.56	0.80	0.43
Body fat percentage	38.62 ± 5.79	39.06 ± 5.51	38.17 ± 6.10	0.90	0.37
SBP (mmHg)	118.15 ± 15.56	121.57 ± 13.42	114.64 ± 16.95	1.15	0.14
DBP (mmHg)	72.85 ± 10.90	73.76 ± 9.32	71.92 ± 12.38	0.25	0.80
FPG (mmol/l)	4.86 ± 1.30	4.78 ± 1.09	4.94 ± 1.50	− 0.49	0.62
TG (mmol/l)	0.87 (0.73)^‡^	0.94 (0.68)^‡^	0.82 (0.73)^‡^	629.5^ǂ^	0.55
LDL (mmol/l)	3.14 ± 0.85	2.98 ± 0.82	3.30 ± 0.87	− 1.75	0.08
HDL (mmol/l)	1.43 ± 0.32	1.44 ± 0.37	1.42 ± 0.27	0.23	0.82
Total cholesterol (mmol/l)	5.06 ± 0.94	4.91 ± 0.85	5.21 ± 1.02	− 1.42	0.16

*Kg* Kilogram, *cm* centimeter, *BMI* Body mass index, *m*^2^ square meter, *SBP* Systolic blood pressure, *mmHg* Millimeter mercury, *DBP* Diastolic blood pressure, *FPG* Fasting plasma glucose, *TG* Triglyceride, *LDL* Low density lipoprotein, *HDL* High density lipoprotein, *l* Littre

^‡^Median and IQR were shown due to lack of normality in distribution

^ǂ^Mann–Withney U test was performed for the comparison and Mann–Whithney U value was presented in the table

Nutrition and physical activity knowledge, attitude and practice of subjects as per intervention and control groups at baseline and 6th month of intervention are presented in Table 2. Table 3 shows the summary of repeated measures ANOVA for nutrition knowledge, attitude, practice, EAT-26 score and TFEQ-18 score as well as physical activity knowledge, attitude, practice and IPAQ score. The result revealed that the within subjects’ effect of repeated measures of test for nutrition knowledge 1 score was statistically significant (f = 34.11, *p* < 0.001). The main effect of group was statistically significant for nutrition knowledge-1 score (f = 6.02, *p* = 0.02) and nutrition attitude (f = 0.43, *p* = 0.01). The interaction between group and time was significant for nutrition knowledge 1 score (f = 6.02, *p* = 0.02).

**Table 2:** Description and comparison of nutrition knowledge, attitude and practice of study subjects as per intervention and control groups at baseline and 6th month of study

		Intervention n = 34		Control n = 35	
		Before	After	Before	After
Nutrition knowledge 1		20.00 ± 3.00	22.59 ± 1.92	19.26 ± 2.64	20.31 ± 2.54
Nutrition knowledge 2		8.41 ± 2.00	9.00 ± 0.85	8.43 ± 0.98	8.43 ± 0.85
Nutrition attitude		29.44 ± 5.41	29.09 ± 3.68	29.77 ± 4.16	30.03 ± 2.75
Nutrition practice		17.44 ± 1.73	17.41 ± 1.28	17.60 ± 2.33	17.89 ± 2.23
EAT-26 score		16.03 ± 9.52	18.76 ± 10.83	15.34 ± 8.44	15.54 ± 10.12
TFEQ-18	Cognitive resistant	14.59 ± 3.03	15.26 ± 3.02	14.77 ± 2.60	15.14 ± 2.77
	Uncontrolled eating	23.56 ± 3.77	23.62 ± 3.45	23.17 ± 3.46	24.46 ± 3.62
	Emotional eating	10.12 ± 1.63	10.35 ± 1.79	9.89 ± 1.98	9.83 ± 2.01
	Total score	48.26 ± 6.19	49.23 ± 5.97	47.83 ± 6.35	49.43 ± 6.32
Physical activity knowledge		6.97 ± 1.68	7.18 ± 1.60	7.23 ± 1.26	7.37 ± 1.35
Physical activity attitude		19.47 ± 7.69	18.53 ± 5.49	20.54 ± 5.07	19.43 ± 4.62
Physical activity practice		15.82 ± 3.63	14.68 ± 2.51	18.11 ± 3.60	17.89 ± 3.43
IPAQ score		1413.00 (2665.50)^‡^	2446.50 (2863.50)^‡^	847.50 (1193.75)^‡^	613.20 (1498.00)^‡^

*EAT-26* Eating attitude test-26, *TFEQ-18* Three factor eating questionnaire-18, *IPAQ* International physical activity questionnaire

^‡^Median and IQR were shown

**Table 3:** Summary of repeated measures ANOVA for Nutrition knowledge, attitude and practice of study subjects

		Time		Group		Time*group	
		f	*p*	f	*p*	f	*p*
Nutrition knowledge 1		34.11	< 0.001**	6.02	0.02*	6.02	0.02*
Nutrition knowledge 2		2.40	0.13	1.36	0.25	2.40	0.13
Nutrition attitude		0.01	0.93	0.43	0.01**	0.28	0.60
Nutrition practice		0.28	0.59	0.62	0.43	0.43	0.51
EAT-26 score		1.77	0.19	0.89	0.35	1.32	0.25
TFEQ-18	Cognitive resistant	2.75	0.10	0.003	0.96	0.80	0.63
	Uncontrolled eating	3.16	0.08	0.08	0.77	2.63	0.11
	Emotional eating	0.14	0.71	0.98	0.32	0.39	0.54
	Total score	3.77	0.06	0.01	0.93	0.23	0.64
Physical activity knowledge		0.48	0.49	0.70	0.41	0.02	0.87
Physical activity attitude		2.19	0.14	0.65	0.42	0.02	0.90
Physical activity practice		6.75	0.01**	16.00	< 0.001**	1.62	0.21
IPAQ score		4.09	0.04*	6.12	0.02*	11.94	0.01**

*EAT-26* Eating attitude test-26, *TFEQ-18* Three factor eating questionnaire-18

*Significant at α = 0.05

**Significant at α = 0.01

Table 4 showed the Bonferroni post-hoc test for the comparison of the nutrition knowledge, attitude and practice as well as EAT-26 score and total and subscale scores of TFEQ and physical activity knowledge, attitude and practice as well as IPAQ score at baseline and 6^th^ month between intervention and control groups. Significant difference was only observed in nutrition knowledge 1 score (*p* < 0.001) and nutrition knowledge 2 score (*p* = 0.01) at 6^th^ month of intervention between intervention and control groups. All physical activity variables were not significantly different at baseline between intervention and control groups except for physical activity practice score (*p* = 0.01). Significant difference was observed in physical activity practice score (*p* < 0.001) and IPAQ score (*p* = 0.02) at 6th month of intervention between intervention and control groups.

**Table 4:** Comparison of nutrition knowledge, attitude and practice as well as EAT-26 and TFEQ-18 score of study subjects between intervention and control groups at baseline and 6th month of study

		Time	Group (I)	Group (J)	Mean difference (I–J)	*p*	η^2^
Nutrition knowledge 1		Baseline	Intervention	Control	0.74	0.28	0.02
		6th month	Intervention	Control	2.27	< 0.001**	0.21
Nutrition knowledge 2		Baseline	Intervention	Control	− 0.02	0.96	0.00003
		6th month	Intervention	Control	0.57	0.01**	0.10
Nutrition attitude		Baseline	Intervention	Control	− 0.33	0.77	0.001
		6th month	Intervention	Control	− 0.94	0.23	0.02
Nutrition practice		Baseline	Intervention	Control	0.16	0.75	0.002
		6th month	Intervention	Control	− 0.47	0.28	0.02
EAT-26 score		Baseline	Intervention	Control	0.69	0.75	0.002
		6th month	Intervention	Control	3.22	0.21	0.02
TFEQ-18	Cognitive resistant	Baseline	Intervention	Control	− 0.18	0.79	0.001
		6th month	Intervention	Control	0.12	0.86	0.0005
	Uncontrolled eating	Baseline	Intervention	Control	0.39	0.66	0.003
		6th month	Intervention	Control	− 0.84	0.33	0.01
	Emotional eating	Baseline	Intervention	Control	0.23	0.60	0.004
		6th month	Intervention	Control	0.52	0.26	0.02
	Total score	Baseline	Intervention	Control	0.44	0.77	0.001
		6th month	Intervention	Control	− 0.19	0.90	0.0002
Physical activity knowledge		Baseline	Intervention	Control	− 0.29	0.43	0.01
		6th month	Intervention	Control	− 0.23	0.53	0.01
Physical activity attitude		Baseline	Intervention	Control	− 1.07	0.41	0.01
		6th month	Intervention	Control	− 0.90	0.46	0.01
Physical activity practice		Baseline	Intervention	Control	− 2.08	0.01**	0.09
		6th month	Intervention	Control	− 3.16	< 0.001**	0.21
IPAQ score		Baseline	Intervention	Control	453.80	0.47	0.01
		6th month	Intervention	Control	3277.97	0.02*	0.13

*EAT-26* Eating attitude test-26, *TFEQ-18* Three factor eating questionnaire-18

*Significant at α = 0.05

**Significant at α = 0.01

## Discussion

In the current study, a total of 85 subjects with confirmed diagnosis of PCOS participated in the study. Overall 16 subjects (18.8%) dropped out from the study among which 9 subjects dropped from the initial 43 subjects in the intervention group (20.9%) and 7 subjects dropped from the initial 42 subjects in the control group (16.7%).

In the current study, the nutritional education significantly increased nutritional knowledge of the subjects compared to the control group regardless of high baseline levels of nutrition knowledge in both groups. Furthermore, the findings of the current study revealed that 6 months of intervention could not increase nutrition attitude and practice of the subjects in intervention group compared with the control group. The nutrition attitude and practice of the subjects were also high at baseline in both groups. These finding was similar to the findings of an eight-week intervention to increase fruit intakes on 104 healthy adults with normal BMI that significantly improved nutrition knowledge but could not increase nutrition attitude and practice [29].

The current study found that the intervention resulted in reduced weight, BMI, and markers of abdominal obesity. These findings were in line with the findings of previous studies [30–32]. Various studies have been published regarding the effects of lifestyle interventions on anthropometric measurements in PCOS and reported promising results; however majority of the studies did not include a holistic, multidimensional intervention [30–32]. Furthermore, due to the heterogeneity in terms of theory methodology and assessment tools, the synergistic effects of diet, exercise and stress management interventions could not be compared between previous studies and the current study.

Although the intervention resulted in a statistically significant reduction in anthropometric parameters among PCOS women, no statistically significant reduction was observed in terms of lipid profile and FPG. Previous studies have shown that lifestyle interventions, including diet or exercise, significantly reduced LDL, TG and total cholesterol, while interventions that included exercise component significantly increased HD [33–35]. A reason for the difference in the findings of current study and previous study was that the current study only included tips and education on healthy eating and no personalized calorie restriction was administered to the subjects. Furthermore, the level of physical activity in the intervention was moderate, therefore, the effects of the intervention on serum lipids and FPG did not reach statistical significance. Another reason for the current observation could be the small number of subjects with abnormal lipid profile and FPG in the current study.

The current study found no significant difference in terms of EAT-26 and TFEQ scores between intervention and control groups as well as within each group from baseline to the end of the study. Danielsen et al. reported that, an intensive exercise program for obese individuals resulted in no improvements in eating behaviour (TFEQ score) at 6th months of intervention [36]. However, there was improvements in eating behaviour (TFEQ score) after one year of intervention. This finding indicates that longer duration of intervention might be required in order to observe a significant improvement in eating behaviour of obese subjects.

The current study reported no significant increase in physical activity knowledge and attitude in the intervention group compared with the control group. Although the physical activity practice score of the subjects in both groups were not significantly different, the IPAQ score of the subjects in intervention group significantly increased after the intervention. This inconsistency in results might be due to over-reporting of physical activity by control group, as evidenced by high physical activity practice scores and low IPAQ scores in control group, and neglecting exercise performance at intervention sessions by subjects in the intervention group, as evidenced by disproportionate higher IPAQ scores in spite of physical activity practice scores in intervention group. In a systematic review in 2012, physical activity intervention resulted in a significant increase in physical activity knowledge of school-aged subjects but the effect on physical activity attitude was negative and more prominent in control group [37].

This study had some limitation. Firstly, we were unable obtain blood test which is required to PCOS and metabolic syndrome due to financial constraint. Secondly, ultrasound scan was only limited to women who were suspected to have PCOS. Thirdly, on physical activity and nutrition knowledge, attitude and practice was self-reported by the, which increases the risk of bias due to over or under-reporting. It is recommended for further researchers to assess physical activity of the subjects using objective methods including pedometers as well as other nutritional biomarkers in further studies.

## Conclusions

Our study found that there was improvement of mean score for some aspects of both nutrition and physical activity albeit not all improvement was significant. This health education may be useful in guiding Malaysian women with PCOS to improve their knowledge, attitude and practise of diet and physical activity. However, there is more to be explore on the effect of this health education of biochemical and anthropometric parameter as well as the optimum length of intervention.

## Data Availability

The datasets generated and/or analysed during the current study are not publicly available due to university rules and regulation of data ownership but are available from the corresponding author on reasonable request.

## Abbreviations

PCOS: Polycystic ovarian syndrome
KAP: Knowledge, attitude and practise
CVD: Cardiovascular disease
GAGS: Global acne grading system
O&G: Obstetrics and gynaecology
PCO: Polycystic ovarian
mm: Millimetre
nmol/L: Nanomoles per litre
ng/dL: Nanograms per decilitre
IPAQ: International physical activity questionnaire
MET: Metabolic equivalents
MET min/w: Minutes per week
KMO: Kaiser–Meyer–Olkin
Kg: Kilogram
cm: Centimeter
BMI: Body mass index
m^2^: Square meter
SBP: Systolic blood pressure
mmHg: Millimeter mercury
DBP: Diastolic blood pressure
FPG: Fasting plasma glucose
TG: Triglyceride
LDL: Low density lipoprotein
HDL: High density lipoprotein
l: Littre
EAT-26: Eating attitude test-26
TFEQ-18: Three factor eating questionnaire-18
